# Angiotensin-Converting Enzyme Gene Polymophism in Adult Primary Focal Segmental Glomerulosclerosis

**DOI:** 10.14740/jocmr1550w

**Published:** 2014-05-22

**Authors:** Rozita Mohd, Zaimi Abdul Wahab, Rizna Cader, Halim A. Gafor, Azizah Md Radzi, Shamsul Azhar Shah, Norella Kong Chiew Tong

**Affiliations:** a Department of Medicine, Pusat Perubatan Universiti Kebangsaan Malaysia (PPUKM), Jalan Yaacob Latiff, Bandar Tun Razak, 56000 Cheras, Kuala Lumpur, Malaysia; b Institute of Medical Research, Malaysia; c Department of Community Health, Pusat Perubatan Universiti Kebangsaan Malaysia (PPUKM), Jalan Yaacob Latiff, Bandar Tun Razak, 56000 Cheras, Kuala Lumpur, Malaysia

**Keywords:** Primary focal segmental glomerulosclerosis, ACE gene polymorphism, Urine protein creatinine index, Estimated glomerular filtration rate

## Abstract

**Background:**

Primary focal segmental glomerulosclerosis (FSGS) accounts for a third of biopsy-proven primary glomerulonephritis in Malaysia. Pediatric studies have found the insertion/deletion (I/D) polymorphism of the angiotensin-converting enzyme (ACE) gene to be associated with renal disease progression. The aim of this study was to determine the prevalence of the ACE (I/D) genotypes in adult primary FSGS and its association with renal outcome on follow-up.

**Methods:**

Prospective observational study involving primary FSGS patients was conducted. Biochemical and urine tests at the time of study were compared to the time of the diagnosis and disease progression analyzed. ACE gene polymorphism was identified using polymerase chain reaction amplification technique and categorized into II, ID and DD genotypes.

**Results:**

Forty-five patients with a median follow-up of 3.8 years (interquartile range: 1.8 - 5.6) were recruited. The commonest genotype was II (n = 23, 51.1%) followed by ID (n = 19, 42.2%) and DD (n = 3, 6.7%). The baseline characteristics were comparable between the II and non-II groups at diagnosis and at study recruitment except that the median urine protein-creatinine index was significantly lower in the II group compared to the non-II group (0.02 vs. 0.04 g/mmol (P = 0.03). Regardless of genotypes, all parameters of renal outcome improved after treatment.

**Conclusion:**

The II followed by ID genotypes were the predominant ACE gene alleles in our FSGS. Although the D allele has been reported to have a negative impact on renal outcome, treatment appeared to be more important than genotype in preserving renal function in this cohort.

## Introduction

The incidence of focal segmental glomerulosclerosis (FSGS) has been reported to be between 5% and 20% of biopsies in adults presenting with the nephrotic syndrome [1]. In a retrospective review of some 1,000 renal biopsies performed at our own center from 1987 to 2007, primary FSGS was the second most common diagnosis after IgA nephropathy for primary glomerulonephritis accounting for 33.1% of all cases [2]. In the second report of the Malaysian Registry of Renal Biopsy (MRRB), FSGS was the commonest primary glomerulonephritis in the < 15 years age group (34%) and the second after minimal change disease in the 15 - 25 years age group [3].

Overall renal survival has been reported to be 45% and 33% at 5 and 10 years respectively for patients who did not attain remission [4]. In the United States, the annual incidence of end stage renal disease (ESRD) attributed to primary FSGS had increased 11-fold from 0.2% to 2.3% between 1980 and 2000, making it the most common cause of ESRD due to primary glomerular disease [1].

The angiotensin-converting enzyme (ACE) gene is located on the long arm of chromosome 17q23 and its main product is ACE. Polymorphism of this gene involves the presence (insertion, I) or absence (deletion, D) of a 287-bp sequence of DNA in intron 16 of the gene resulting in the genotypes II, ID or DD. Polymorphism of the ACE gene was thought to influence disease progression as homozygosity for the deletion (D) polymorphism is associated with higher levels of angiotensin II (AII) [4, 5]. Higher levels of AII result in many adverse renal effects which include proliferation of mesangial cells and increase in mesangial matrix, expression of TGFβ and platelet-derived growth factor. All these promote collagen accumulation and eventually lead to renal fibrosis [4].

ACE gene polymorphism has been investigated as one of the genetic factors that may affect the progression of a variety of renal diseases (for example, diabetic nephropathy, ESRD, IgA nephropathy) [6-9]. In contrast, there are little data regarding the role of ACE (I/D) genotypes in adults with primary FSGS. Studies in the pediatric population have shown the DD genotype to be more frequent in FSGS than in minimal change disease or control patients [10-12]. Affected patients tended to present with clinical symptoms at an earlier age, to be steroid-resistant and to have a higher incidence of chronic renal failure compared to those with other genotypes. Whereas studies in adult population reported conflicting results. As there are no such data available in adult Malaysian patients with FSGS and the influence of these polymorphisms on disease outcome, we embarked on this study to determine the prevalence of ACE gene polymorphism in our adult FSGS patients and to evaluate whether this impacted on disease outcome on follow-up (at study recruitment).

## Materials and Methods

This was a prospective observational study in biopsy-proven primary FSGS patients attending the Nephrology Clinic at our institution. The diagnosis of primary was made after secondary causes of FSGS were excluded. We evaluated the amount of proteinuria and estimated glomerular filtration rate (eGFR) at initial presentation and at study recruitment as these two parameters would determine the patients’ clinical response, namely complete remission, partial remission or no remission and their renal status respectively. Proteinuria was quantified using the morning spot urine protein-creatinine index (uPCI) which has been validated [13]. A value of ≤ 0.02 g/mmol was normal.

The eGFR was calculated using the modification of diet in renal disease calculator for patients 18 years of age and older. The Schwartz formula was used for patients below the age of 18 years. These parameters were used to define the renal status which was then classified according to the chronic kidney disease (CKD) staging severity based on the NKF-K/DOQI guidelines [14]. The presence of co-morbidities which might influence renal function was also documented. Medications used, in particular steroids and other immunosuppressive drugs, were recorded. This study was approved by the Research and Ethics Committee of our institution and funded by the Research Grant of the same institution (FF-237-2008).

### Genetic identification

Five milliliters of blood was collected in an EDTA bottle and kept at -4 °C (< 48 h) for DNA extraction using a commercial DNA extraction kit (Qiagen kit) for purity and reliability. The DNA samples extracted were processed using polymerase chain reaction technique. Using a set of primers CTG GAG ACC ACT CCC ATC CTT TCT and GAT GTG GCC ATC TTC GTC AGA T, the DNA was denaturated following a specific protocol. Fragments with deletion (D allele) and with insertion (I allele) of 190 bp and 490 bp respectively were detected on 1% agarose gel with ethidium bromide. Since 4-5% of ID genotype can be misclassified as DD when only a flanking primer pair is used, the DNA samples were processed twice using differrent primer sets TGG GAC CAC AGC GCC GCG CAC TAC and TCG CCA GCC CTC CCA TGC CCA TAA to increase the specificity of the DD genotyping. Only the I allele produces a 335 bp amplification and was identified on a 1.5% agarose gel containing ethidium bromide. The reaction yielded no products in the samples of DD genotype.

### Statistical analysis

Data were analyzed using the SPSS 15 software. All data were expressed as median with interquartile range (IQR) since data were not normally distributed. The Wilcoxon and Mann-Whitney U tests were used for numerical data chi-square or Kruskal-Wallis was used for categorical data.

## Results

Forty-five patients had positive genotype results. These comprised 30 males (66.7%) and 15 females (33.3%). There were 29 Malays (64.4%), 15 Chinese (33.3%) and one Indian (2.2%) reflecting their proportions in the country.

Their median age at the time of diagnosis was 30 years (range: 14 - 71 years). The majority of patients (44.4%, n = 20) were in the 15 - 30 age group, 20% (n = 9) in the 30 - 45 age group and 28.9% (n = 13) above 45 years. As this was an adult population, only three (6.7%) patients were under 15 years old. Their median age at the time of study recruitment was 38 years (IQR: 24 - 51, range: 17 - 91). The median duration of follow-up was 3.8 years (1.8 - 5.6).

The commonest clinical presentation was nephrotic syndrome with or without renal failure (n = 32, 71.1%) followed by asymptomatic proteinuria (n = 7, 15.5%) and chronic renal failure (n = 6, 13.4%) in descending order. Hypertension was present in 20 (44.4%) of the patients at diagnosis. Eighteen (40%) had no other illnesses whilst seven (15.6%) had unrelated medical illnesses such as bronchial asthma.

The commonest genotype was II (n = 23, 51.1%) followed by ID (n = 19, 42.2%) and finally DD (n = 3, 6.7%). The II made up 51.1% of the study population and the ID/DD formed the other 48.9%.

Both II and ID/DD groups had comparable baseline parameters at diagnosis as shown in Table 1. However, the patients in the II group were younger (27 vs. 33 years old), had lower serum creatinine (87 vs. 93.5 μmol/L) and higher eGFR (88 vs. 63 mL/min/1.73 m^2^) despite having more severe nephrosis with uPCI of 0.56 g/mmol vs. 0.42 g/mmol and serum albumin of 24.4 g/L vs. 25.2 g/L.

**Table 1:** Demographics of the II Group Versus ID/DD Group at Diagnosis

	II^a^ (n = 23)	ID/DD^a^ (n = 22)	P value^b^
Age (years)	27 (21 - 46)	33 (22 - 53)	0.46
Gender (M:F)	17:06	13:09	
Race (M:C:I:O)	14:7:1:1	14:8:0:0	
Systolic BP (mm Hg)	134 (124 - 148)	132 (126 - 150)	0.31
Diastolic BP (mm Hg)	78 (74 - 85)	84 (74 - 92)	0.24
MAP (mm Hg)	99 (90 - 105)	103 (91 - 109)	0.18
Weight (kg)	65.2 (56.8 - 75.4)	67.8 (58 - 73)	0.69
BMI (kg/m^2^)	24.4 (21.3 - 29.2)	25.2 (22.5 - 27.8)	0.69
Serum albumin (g/dL)	22 (16 - 32)	26 (17 - 39)	0.51
Total cholesterol (mmol/L)	7.62 (6.01 - 9.65)	7.48 (5.8 - 12.2)	0.94
TG (mmol/L)	1.84 (1.28 - 2.53)	2.91 (2.26 - 4.29)	0.69
LDL (mmol/L)	4.61 (3.35 - 6.02)	4.69 (3.05 - 8.70)	0.90
eGFR (mL/min/1.73 m^2^)	88 (50 - 139)	63 (45 - 122)	0.57
uPCI (g/mmol) (NR < 0.02)	0.56 (0.37 - 0.88)	0.42 (0.17 - 0.81)	0.21

^a^Median (interquartile range). ^b^Mann-Whitney U test (P < 0.05 is statistically significant). MAP: mean arterial pressure; BMI: body mass index.

At study recruitment, the II group had achieved complete remission whilst the ID/DD was in partial remission (0.02 g/mmol vs. 0.04 g/mmol, P = 0.03). The II group also had a higher eGFR (91 mL/min/1.73 m^2^ vs. 62 mL/min/1.73 m^2^, P = 0.06) at study recruitment which showed a trend towards significance (P = 0.06). Other parameters were comparable between the two groups (Table 2).

**Table 2:** Demographics of the Two Study Group at Study Recruitment

	II^a^ (n = 23)	ID/DD^a^ (n = 22)	P value^b^
Age (years)	31 (23 - 48)	38 (27 - 54)	0.57
Gender (M:F)	17:06	13:09	
Race (M:C:I:O)	14:7:1:1	14:8:0:0	
Systolic BP (mm Hg)	126 (122 - 120)	127 (117 - 138)	0.56
Diastolic BP (mm Hg)	74 (68 - 81)	80 (67 - 83)	0.53
MAP (mm Hg)	93 (86 - 100)	93 (85 - 99)	0.97
Weight (kg)	68.0 (52.8 - 75.3)	66.4 (59 - 77)	0.76
BMI (kg/m^2^)	25.3 (21.3 - 29.5)	25.8 (20.5 - 30.1)	0.66
Serum albumin (g/dL)	46 (41 - 47)	43 (40 - 45)	0.17
Total cholesterol (mmol/L)	5.39 (5.16 - 5.99)	5.59 (4.63 - 6.83)	0.89
TG (mmol/L)	1.43 (0.88 - 2.17)	1.74 (1.02 - 3.02)	0.38
LDL (mmol/L)	3.38 (2.34 - 3.90)	2.91 (2.26 - 4.29)	0.82
Creatinine (μmol/L)	80 (64 - 113)	100 (70 - 147)	0.10
eGFR (mL/min/1.73 m^2^)	91 (58 - 127)	62 (42 - 98)	0.06
uPCI (g/mmol)	0.02 (0.01 - 0.04)	0.04 (0.02 - 0.14)	0.03
Duration of follow-up (years)	3.3 (1.5 - 5.8)	4.0 (2.3 - 5.7)	

^a^Median (interquartile range). ^b^Mann-Whitney U.

Almost two-thirds (n = 30, 66.6%) of the patients had experienced a relapse. These included 16 patients (69.5%) from the II group, seven (63.1%) from the ID and two (66.7%) from the DD group. The number of relapses increased with time of follow-up.

In total, 91.1% (n = 41) of the study population were in complete or partial remission at study recruitment. The II group had the highest number of patients with complete remission (n = 18, 64%) followed by the ID (n = 9, 32%) and DD (n = 1, 4%). Four patients who did not achieve remission were from the II (n = 1) and ID (n = 3) groups, suggesting the D allele may predispose to more resistant disease.

Majority of the patients (n = 37, 82.2%) were treated with corticosteroids followed by 2 months of oral oral cyclophosphamide 64.4% (n = 29, 64.4%) and 12- 18 months of ciclosporin A (n = 24, 53.3%). Other immunosuppressive agents used in order of decreasing frequency were azathioprine (n = 12, 26.7%), mycophenolate mofetil (MMF) (n = 6, 13.3%) and tacrolimus (n = 3, 6.7%). Renin-angiotensin-aldosterone inhibitors were used in two-thirds of the patients either as single agent or in combination (n = 34,75.6%), whilst the remaining 11 patients were on neither for reasons such as hyperkalaemia or development of acute on chronic renal failure.

## Discussion

Primary FSGS remains an enigma to the nephrology community due to its complexity and poor response to older therapy. The progression to ESRD within 3 - 5 or 10 years especially in those who are not responding to current mainstay treatment with corticosteroids is a major concern [15-18]. Some studies have shown a racial predominance leading to the belief that genetic factors played an important role in its development and progression [16, 19, 20].

As AII has been hypothesized to be one of the main culprits in the progression of primary FSGS to ESRD [21, 22], polymorphism of the ACE gene was thought to be of clinical relevance. Some studies have shown a positive association between the I/D polymorphism and CKD progression [10, 11, 23]. Since previous studies have focused mainly on children and Caucasians, we therefore conducted a study on our local adult FSGS patients to ascertain the frequency of this polymorphism and to determine its association, if any, with renal outcome on follow-up.

Our population was comprised of 45 patients with a male to female ratio of 2:1 and the median age at diagnosis was 30 years (IQR: 21.5 - 49.0). The predominant male prevalence is almost universal although the age at first diagnosis may vary in other continents. Several studies in Caucasian populations had demonstrated their FSGS patients to be older by some 10 years at presentation and male patients accounted between 58 and 63% [16, 24, 25]. In contrast, our patients presented at a younger age and this may be explained by differences in both ethnic and environmental factors.

In our study, nephrotic syndrome was the commonest presentation with or without renal failure followed by asymptomatic proteinuria and chronic renal failure. In addition, hypertension was present in 44.4% of the patients and generally occurred in the older patients. These findings concur with these of previous studies [3, 16, 24, 25]. Thus the clinical presentation of FSGS in adults is fairly homogenous despite diverse geographical and ethnic backgrounds.

The median duration of follow-up for our patients was 3.8 years (IQR: 1.8 - 5.6). The shortest follow-up was 3 months in three patients and the longest follow-up was 23 years in one patient. This is an acceptable time frame for assessing their renal outcome as many previous studies have done this as early as 2 - 5 years [15, 19, 26].

Most of our patients were treated initially with steroids either orally or by mini pulses of intravenous methylprednisolone followed by oral prednisolone at 0.5 mg/kg/day (usual dose 30 mg/day) and reducing. The most commonly used immunosuppressant was oral cyclophosphamide for 2 months for frequent relapsers or resistant cases and maintenance with ciclosporin A was used. MMF and tacrolimus were only used if patients could not tolerate ciclosporin A or as triple therapy (steroids/calcineurin inhibitor/MMF) in selected resistant cases. These were maintained for 12 - 18 months and slowly weaned. Most patients with resistant or frequently relapsing FSGS were kept on alternate-day minimal dose steroids. The usage of ACE inhibitors and angiotensin receptor blockers (ARBs) were almost equal in our patients. The usual strategies to prevent progression of chronic kidney disease were also employed in all patients especially those with persistent residual proteinuria. As no level I evidence is available to guide therapy [27], these approaches were used as we strongly believe that both induction and maintenance of remissions are of utmost importance in preserving renal function.

Our data showed a good and optimistic renal status on follow-up. FSGS, as discussed earlier, is notoriously difficult to treat to remission and to maintain in remission. Its natural history in unresponsive patients is progression to ESRD over 3 - 5 or 8 years [15]. Comparing our results with those obtained by Chun et al, none of our patients had reached ESRD as opposed to the 53% of his patients who did not attain remission and thus, suffered ESRD at 5 years of follow-up [15]. However 20 of our patients (44.4%) had an eGFR of less than 15 mL/min/1.73 m^2^ at diagnosis. Of this, nine patients subsequently required dialysis support. Interestingly, only four (8.8%) patients had stage IV CKD and none had developed ESRD yet at the time of follow-up. Four of our patients had been on follow-up for more than 10 years and one patient had the longest follow-up of 23 years. This further strengthens the finding from previous studies that achieving complete or partial remission was an important clinical predictor of renal survival in patients with primary FSGS.

The most frequent genotype in our cohort was II, followed by ID and finally the DD genotype. This finding concurs with Luther et al where the II allele was the most frequent polymorphism with 56% prevalence in their 71 adult patients with primary FSGS [23]. In a review of adult FSGS with ESRD (n = 47), Dixit et al reported that 55% (n = 26) had the ID genotype, 25% II (n = 12) and 19% DD (n = 9) respectively [28]. It is worthwhile noting that more than half of Dixit’s patients were heterozygous (ID) for the ACE gene [28]. The predominance of the ID genotype in this ESRD patients suggests that the ID genotype may not be as benign as initially thought. It was well established that those homozygous for the DD genotype had highest mean ACE activity while the I allele was the silencer element in the ID group, but Luther et al reported a significant influence of those with the D allele (ID and ID) with FSGS disease progression [4, 5, 23]. This implies that both the ID and DD genotypes may associated with a poor prognosis for renal survival in adult FSGS patients. Nonetheless, all three studies in adult populations did not show increased frequency of the DD genotype as reported by studies in the pediatric population [10, 12]. These suggest that FSGS in children may be a different disease entity than FSGS in adults. Perhaps, other genes and other non-inherited factors may also be involved in the determination of renal outcome than ACE gene polymorphism.

Therefore we grouped the ID and DD genotypes together to explore the potential deleterious effect of the D allele. There was a trend towards poor renal outcome parameters at follow-up in the DD and ID genotypes. The uPCI was highest in the DD with a median of 0.13 g/mmol (IQR: 0.03 - 0.16), while the eGFR was lowest in the ID group with a median of 59 mL/min/1.73 m^2^ (IQR: 37 - 98). The uPCI did show a trend towards significance when comparison was made amongst the three genotypes with the lowest value in the II group. Surprisingly at presentation, the II group had the most number of patients with severe renal failure but at follow-up, most of them regained excellent renal status. This could be attributed to the fact that they achieved the greatest number of complete remissions (n = 18). Of the total study population, 41 patients (91.1%) were either in complete or partial remission at follow-up reflecting the good response to the maintenance treatment strategies. Nevertheless, half of the patients suffered at least one relapse. Almost 60% of the patients in each genotypic group experienced a relapse. The relapsing nature of FSGS is well described in the literature although most of our patients suffered a relapse as a result of non-compliance with therapy and often in association with an intercurrent upper respiratory tract infection [26, 29]. Intragroup analysis in the II and ID/DD at the time of diagnosis and at study recruitment revealed that parameters related to nephrosis (serum albumin, uPCI and lipid profiles) had improved significantly in both groups at follow-up. Despite the apparent influence of the D allele seen in terms of the uPCI at recruitment, it is still possible to achieve partial or complete remission in this group. Troyanov et al reported that even those in partial remission had a significant renal survival compared to those with no remission [26].

Our study demonstrated that complete and partial remissions are achievable with the added remission-maintenance chemotherapy as well as the use of ACE inhibitor and/or ARBs. Despite follow-up beyond 10 years in four patients, none of the 45 patients has developed ESRD to date although we do have some patients with CKD stages III and IV. Nonetheless, early presentation of FSGS before irreversible chronic renal damage has occurred is vital for prolonged renal survival. Patient compliance with prescribed therapy is another major determinant of whether they remain in complete or partial remission.

### Conclusion

The II followed by ID genotypes were the predominant ACE gene alleles in our patients. This pattern is similar to those reported in the literature. The D allele appeared to impact renal outcome in our study population. Primary FSGS is an immune glomerulonephropathy due to T-cell dysregulation and is highly resistant to steroids-only therapy. However, given the armamentarium of immunosuppressive agents now available, the judicious use of low-dose maintainance steroids in conjunction with selected immunosuppressives and the RAS blockers, the natural history of this disease can perhaps be rewritten. Given these findings, routine testing of the ACE gene polymorphism is not warranted in current clinical practice.
